# The ultrasound-guided percutaneous release technique for De Quervain's disease using an acupotomy

**DOI:** 10.3389/fsurg.2022.1034716

**Published:** 2023-01-06

**Authors:** Yifeng Shen, Qiaoyin Zhou, Xiaojie Sun, Zuyun Qiu, Yan Jia, Shiliang Li, Weiguang Zhang

**Affiliations:** ^1^ College of Traditional Chinese Medicine, Fujian University of Traditional Chinese Medicine, Fuzhou, China; ^2^ Urology Department, Hospital of Chengdu University of Traditional Chinese Medicine, Chengdu, China; ^3^ Key Laboratory of Orthopedics & Traumatology of Traditional Chinese Medicine and Rehabilitation, Ministry of Education, FuJian University of TCM. Fuzhou, China; ^4^ Department of Acupuncture and Moxibustion, China-Japan Friendship Hospital, Beijing, China; ^5^ Traditional Chinese Medicine Department, Beijing Jishuitan Hospital, Beijing, China; ^6^ Health Science Center, Peking University, Beijing, China

**Keywords:** de quervain's disease, tendonitis, acupotomy, ultrasound guidance, anatomy

## Abstract

**Background:**

This study aimed to compare the effectiveness and safety of the percutaneous first extensor compartment performed by acupotomy procedure with or without ultrasonic (US) guidance.

**Methods:**

The percutaneous release was performed with an acupotomy on 40 wrists of cadavers, which was divided into US guidance operation and blind operation. Each arm was dissected and assessed regarding the amount of release and the extent of neurovascular and tendon injury. An analysis of finite biomechanical elements based on wrists specimen data is analyzed to observe the stress of the first extensor tendon compartment. A prospective study observed the pain visual analogue score(VAS) and Patient-Rated Wrist Evaluation (PRWEB) changes after the ultrasound guidance or blind acupotomy treatment in 30 dQD patients.

**Results:**

The success rate in the ultrasound-guided technique was 85%, and the blind technique was 70% in the cadaver study, both techniques without neurovascular injury. There was no statistically significant difference between the two groups in measuring the distance from the incision marks to the blood vessels and nerves (*P* > 0.05). According to the biomechanical analysis, the tendon friction rubs when the wrist is upright. When the wrist is flexed, the tendon and tendon sheath is stressed in the bone ridges. In this prospective study, both ultrasound guidance and blind acupotomy treatment achieved well improvements in pain and function (*P* < 0.05), but the results with no statistically significant between groups (*P* > 0.05).

**Conclusion:**

Both blind and US-guided percutaneous release by acupotomy of the first extensor tendon compartment can get a good result. US-guided techniques can improve the success rate during acupotomy operations, especially for beginners and followers.

## Introduction

De Quervain's disease (dQD) is stenosing tenosynovitis of the abductor pollicis longus (APL) and extensor pollicis brevis (EPB) in the first extensor compartment of the wrist, due to the imbalance between the tendons of the first extensor compartment (APL and EPB) and the osteofibrous tunnel. It usually affects women, especially new moms who get “baby wrist.” It has been hypothesized that internal degenerative processes with thickening of the tendon sheath (1), rather than extrinsic inflammatory ones, which are more likely to cause dQD (2). Its treatments include local steroid injection, endoscopic and open surgery (3).

Percutaneous release technology has become more widely used as a minimally invasive technique in the surgical treatment of common ailments in trigger finger (4), carpal tunnel (5), cubital tunnel (6), Dupuytren contracture (7). Research demonstrates that the first annular pulley's percutaneous release procedures produce outcomes comparable to those of open surgery in the trigger finger (8). According to three studies, dQD may be treated efficiently and safely using percutaneous release technology (9–11).

**Figure 1:**
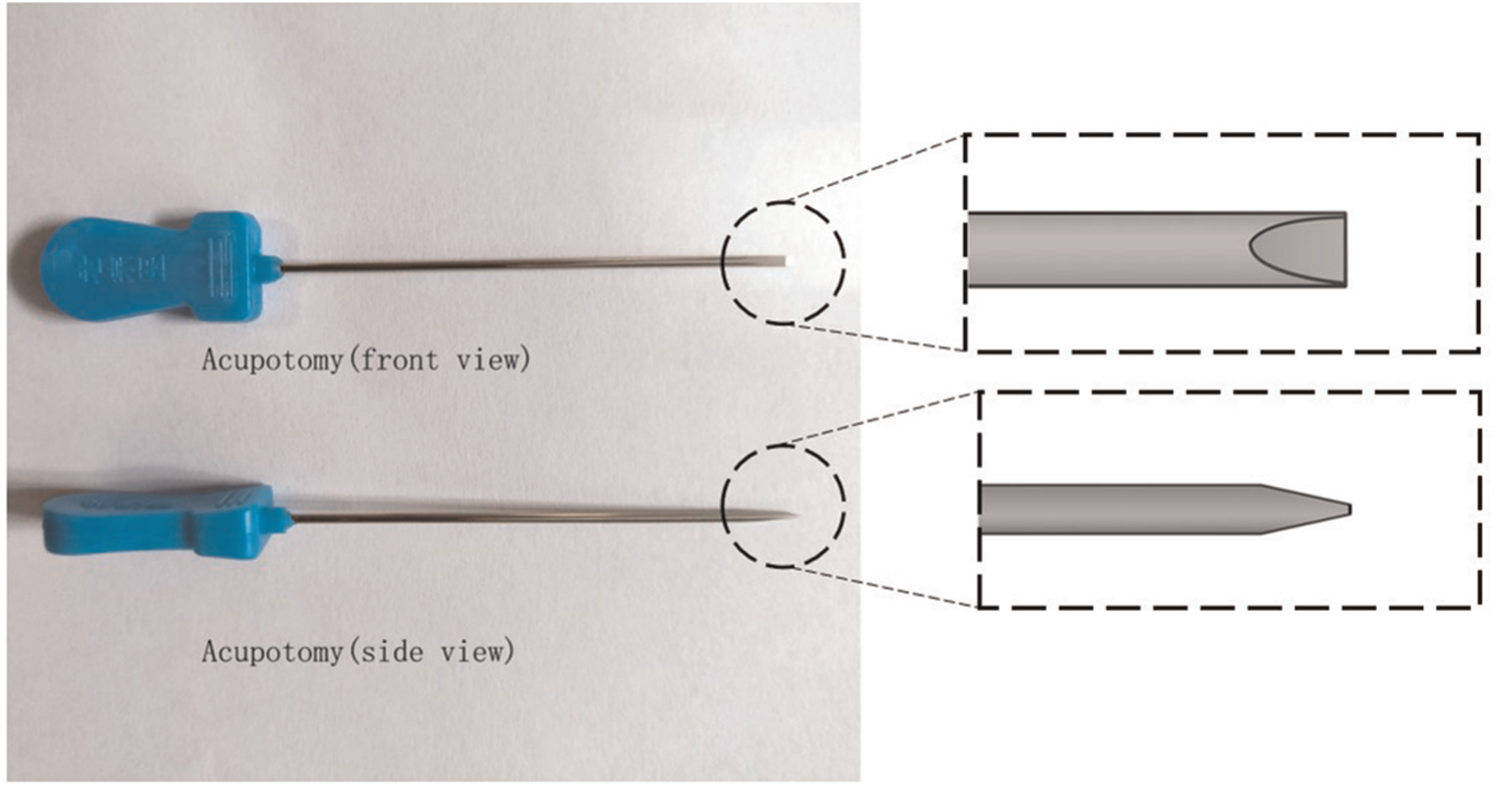
Schematic diagram of the acupotomy.

**Figure 2:**
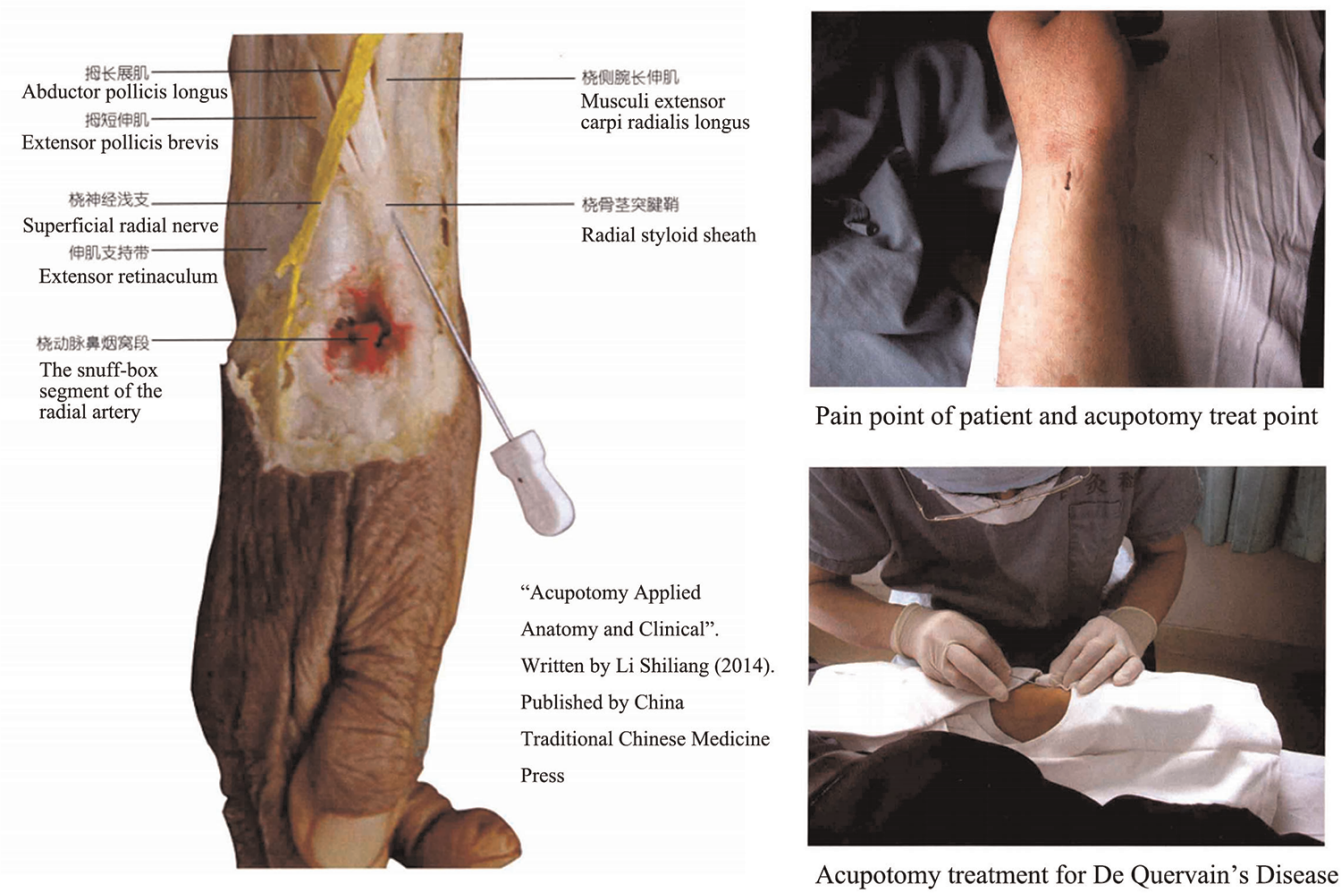
Chapter of De quervain's disease in “Acupotomy Applied Anatomy and Clinical”.

Acupotomy, also named Needle-Knife, consists of a handle, needle body and blade (Figure 1). Traditional Chinese medicine doctors frequently employ acupotomy in their clinical practice, especially in orthopedics and pain management (12). Unlike acupuncture treatment, which stimulates acupoints by rotating and twisting muscles or electrical currents, acupotomy cuts muscle fascia or tendon sheath tissue through its sharp, flat needle tip. However, the classic acupotomy procedure is performed blindly without ultrasonography (US), necessitating a high level of anatomical understanding from the operator and perhaps posing certain operational dangers (13). In the previous research, we assessed the efficacy and safety of ultrasound-guided percutaneous A1 pulley release with acupotomy, the result indicated that the length and percentage of released A1 pulley is longer by ultrasound-guided percutaneous release than performed blindly. In order to treat dQD, the standard clinical practice and anatomical finding of acupotomy for dQD were described in our book “Acupotomy Applied Anatomy and Clinical” in 2014 (Figure 2). This study assessed the accuracy and safety of ultrasound-guided and nonultrasound acupotomy and proposed the anatomical reference in dQD.

## Materials and methods

### Acupotomy release procedure

**Figure 3:**
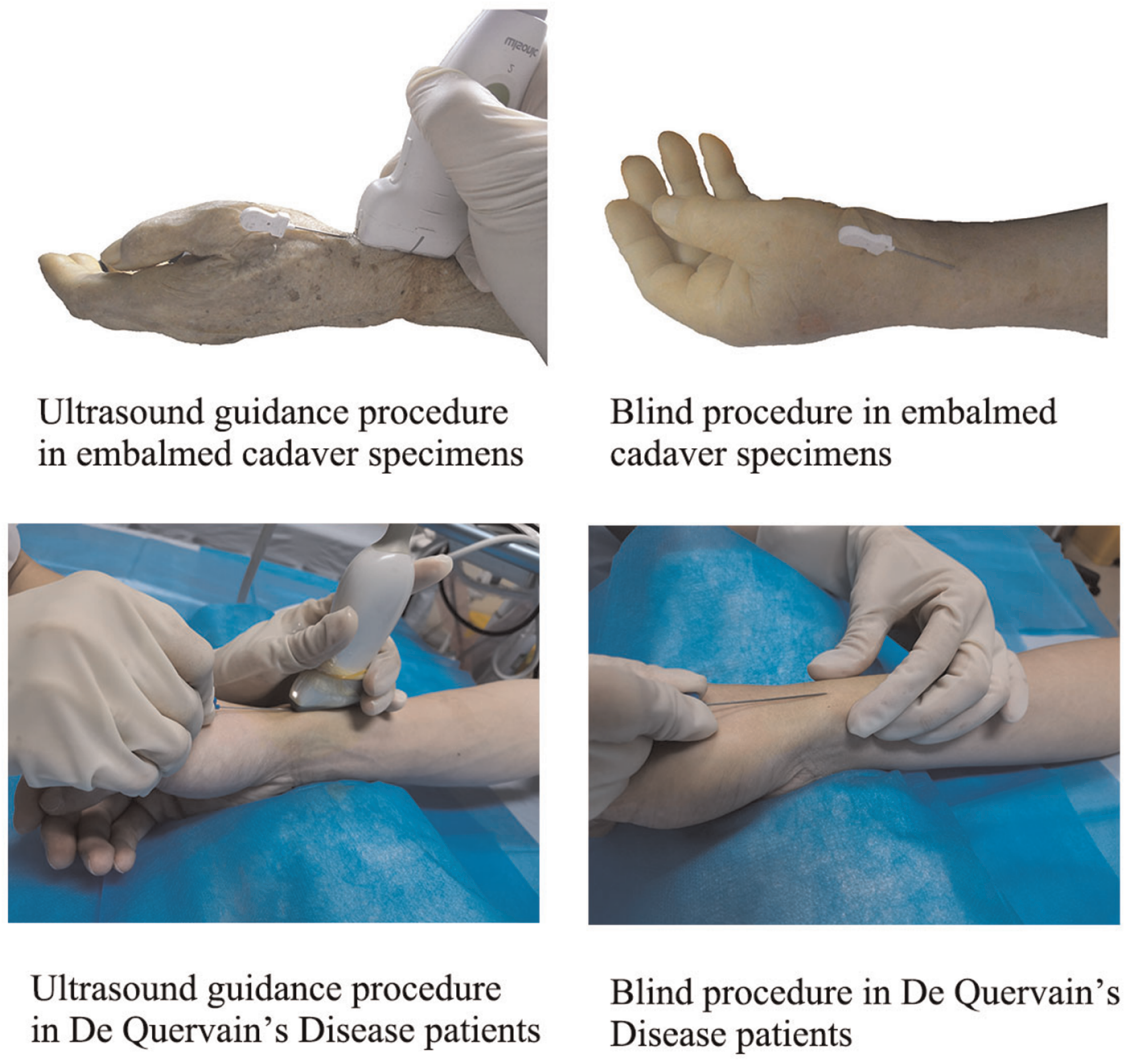
Ultrasound-guided acupotomy procedure and blind acupotomy procedure.

Ultrasound guidance procedure: The arms were placed in a neutral position, and the wrist was kept ulnar to facilitate needle insertion. The high-frequency ultrasound probe was placed on the radial styloid process's surface, and the wrist's first compartment was identified by transverse scanning. The needle is inserted in the plane in skin with an angle of 15°, and the knife surface is perpendicular to the skin. The ultrasound image shows that when the acupotomy penetrates the skin and reaches the surface of the tendon sheath, it is pushed and released once, and it can be clearly felt during the pushing and cutting process. There is a feeling that the tough tissue is cut under the acupotomy. The blind procedure: group used the same method to insert the needle without ultrasound assistance. First, the highest point of the radial styloid process was touched by hand for anatomical positioning, and the needle insertion point was located at the distal end of the radial styloid process. It is located between the two bony ridges of the radial styloid process and is flush with the proximal transverse crease of the wrist. The other operations were the same as those in the ultrasound group (Figure 3).

### Anatomical study

Twenty embalmed cadaver specimens were selected without wrist or hand injuries. The Ethics Committee approved this study of the Peking University School of Medicine for human research (FITCM IACUC 2021077). According to the random number table method, the specimens were divided into 20 wrists in the ultrasound group (U Group) and 20 wrists in the blind group (B Group). A fellow (Yifeng Shen) who had trained in the basic operation of ultrasound and acupotomy performed the experiments. The acupotomy with a one mm-diameter and 50.00 mm in length was used. After the percutaneous release process, the needle is retained in the wrists.

The length of the acupotomy incision was measured using an electronic Vernier caliper. The length of the bony bulge of the radial styloid process is considered to be the length that needs to be released, not the entire extensor retinaculum, to avoid dislocation of the tendon. The ratio of acupotomy release length with the radial styloid bony bulge length was calculated to record the release rate, the full release (release rate ≥ 100%), partial release (0% < release rate < 100%) and the number of unreleased cases (release rate = 0%) were recorded. The position of the acupotomy incision was not located on the surface of the first sheathing canal at the radial styloid process or deviated from the target release area record as a miss. The learning curve demonstrates the change in the release success rate of the two techniques.

**Figure 4:**
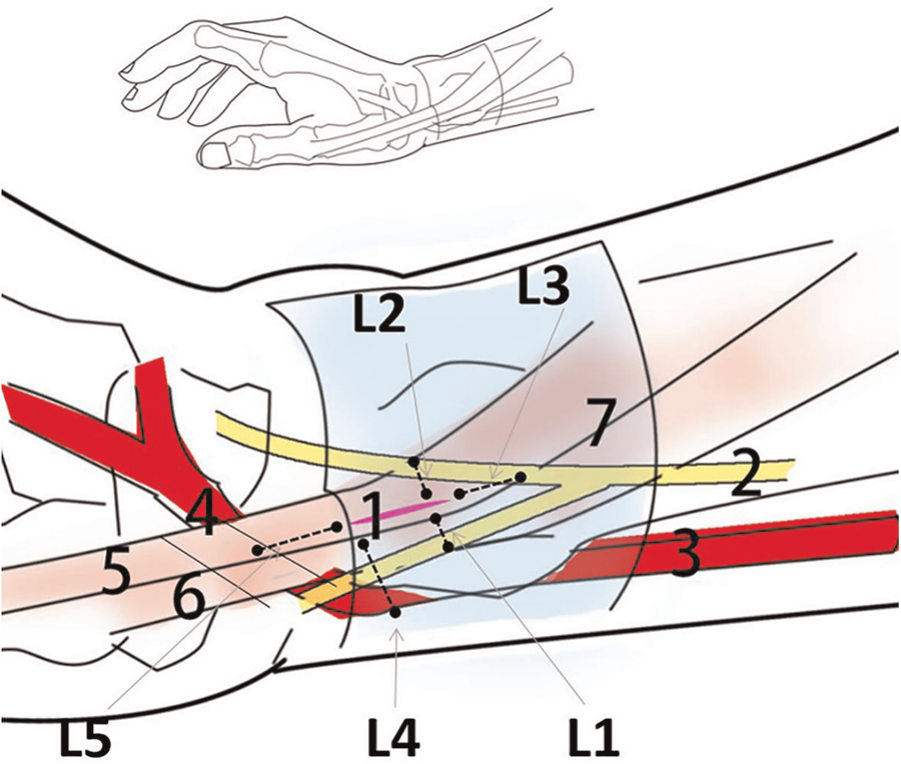
Schematic diagram of the release traces and anatomical structure of the radial styloid process with acupotomy. 1 (Purple line) needle-knife release trace; 2 superficial branch of the radial nerve; 3 radial artery; 4 radial artery snuff fossa; 5 extensor pollicis brevis; 6 abductor pollicis longus; 7 radial wrist retina.

The superficial branch of the radial nerve, radial artery, and tendon were examined for cuts and wounds under a magnifying glass. Tendon injuries were assessed using the following methods: tendon injury (none; minor injury: superficial minor injury or injured tendon thickness <10%; severe injury: tendon rupture or injured tendon thickness >10%). The following distances were measured using an electronic Vernier caliper: the shortest lateral distance (L1) of the incision from the radial branch of the superficial branch of the radial nerve. The incision's shortest lateral distance (L2) from the ulnar branch of the superficial radial nerve. The shortest longitudinal distance from the incision to the bifurcation of the superficial branch of the radial nerve (L3). The shortest lateral distance from the incision to the radial artery (L4) and the shortest longitudinal distance from the incision to the radial artery snuff fossa (L5) (Figure 4).

### Finite element analysis

Modeling was performed by Solidworks software using radial styloid process data measured from 20 human specimens. The model consists of the radial styloid process region's bone tissue, tendon, and tendon sheath. Setting the model's modulus of elasticity, Poisson's ratio, and cross-sectional area for the model's bone, tendon, and tendon sheath according to the literature (14, 15). The axial load was applied to tendon (1) Simulate motion of thumb in the upright position of wrists: apply a force of 50 N to both ends of tendons (2). Simulate the wrist flexion movement: apply a force of 50 N to the proximal end of tendons, apply a force of 30 N to the distal end of tendons, and apply a force of 30 N to the lateral side of the tendons (16). The stress of the radial styloid process area is observed.

### Prospective clinical finding

The prospective clinical study was conducted in the Department of Acupuncture and Moxibustion, The Third People's Hospital Affiliated with the Fujian University of Traditional Chinese Medicine. A 5-year experience doctor (Qiaoyin Zhou) completed the patient's acupotomy operation. Each participant was informed with a consent form. The study comprised patients between 18 and 65 who reported pain, swelling, and tenderness around the radial styloid (VAS ≥ 4 cases, pain duration ≥ 4 weeks) and were identified as having dQD after a positive Finkelstein test. Thirty patients were divided into two groups randomized using a random number table. Group U-P (*n* = 15) received once treatment of ultrasound-guided acupotomy, while Group B-P (*n* = 15) received once therapy of blind acupotomy. A blinded research fellow who was not involved in patient care conducted pre- and post-acupotomy therapy clinical outcome assessments. Before, one week after, and one month after receiving acupotomy therapy, symptoms and functional impairment were evaluated using the visual analogue scale for pain (VAS) and the Patient-rated Wrist Evaluation (PRWE).

### Statistical analysis

All data were analyzed by SPSS 20.0 statistical software. The t-test and Chi-square test was used for comparison between groups, and *P* < 0.05 was considered statistically significant.

## Results

**Figure 5:**
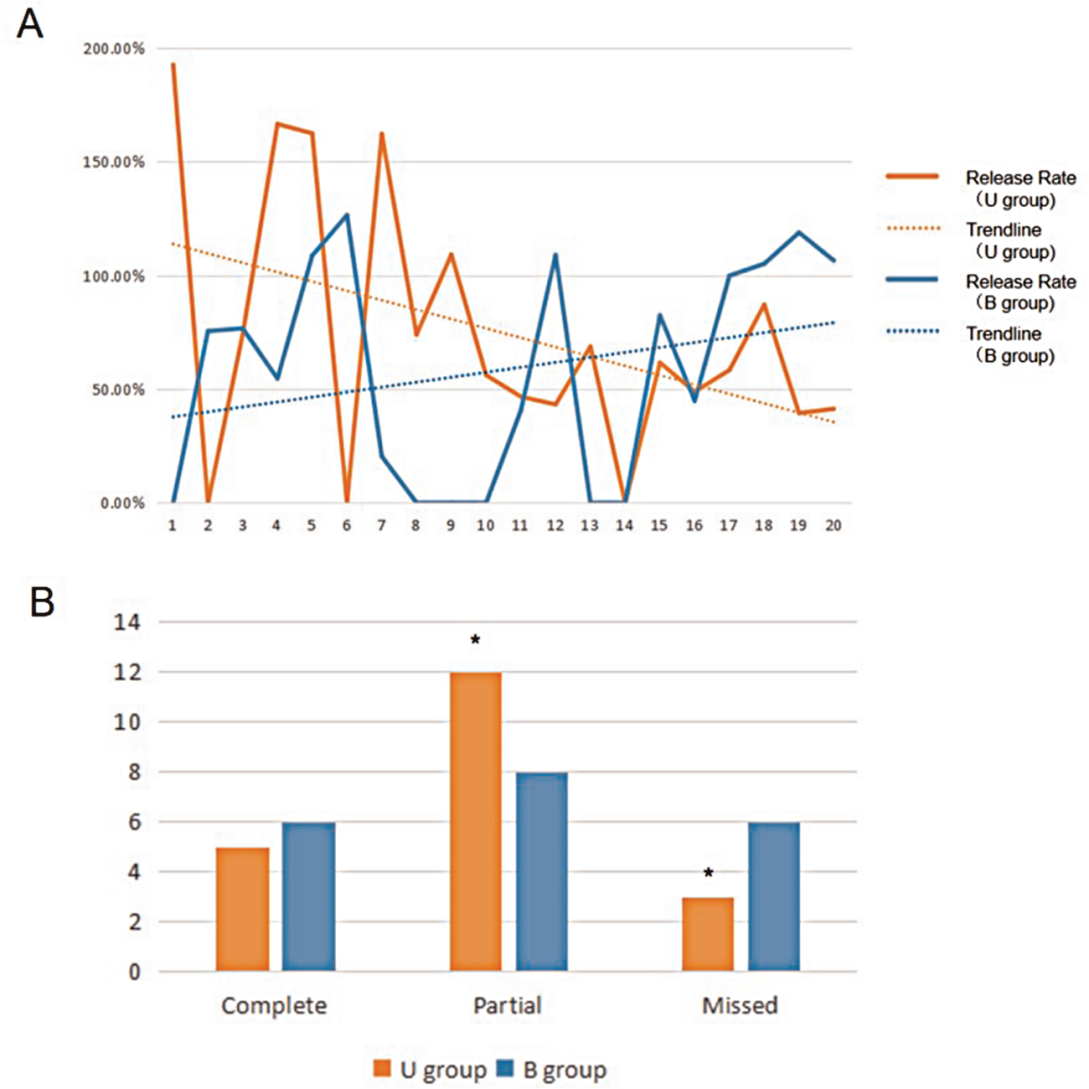
(**A**) Learning curve and (**B**) Percutaneous release situation in U group and B group.

**Figure 6:**
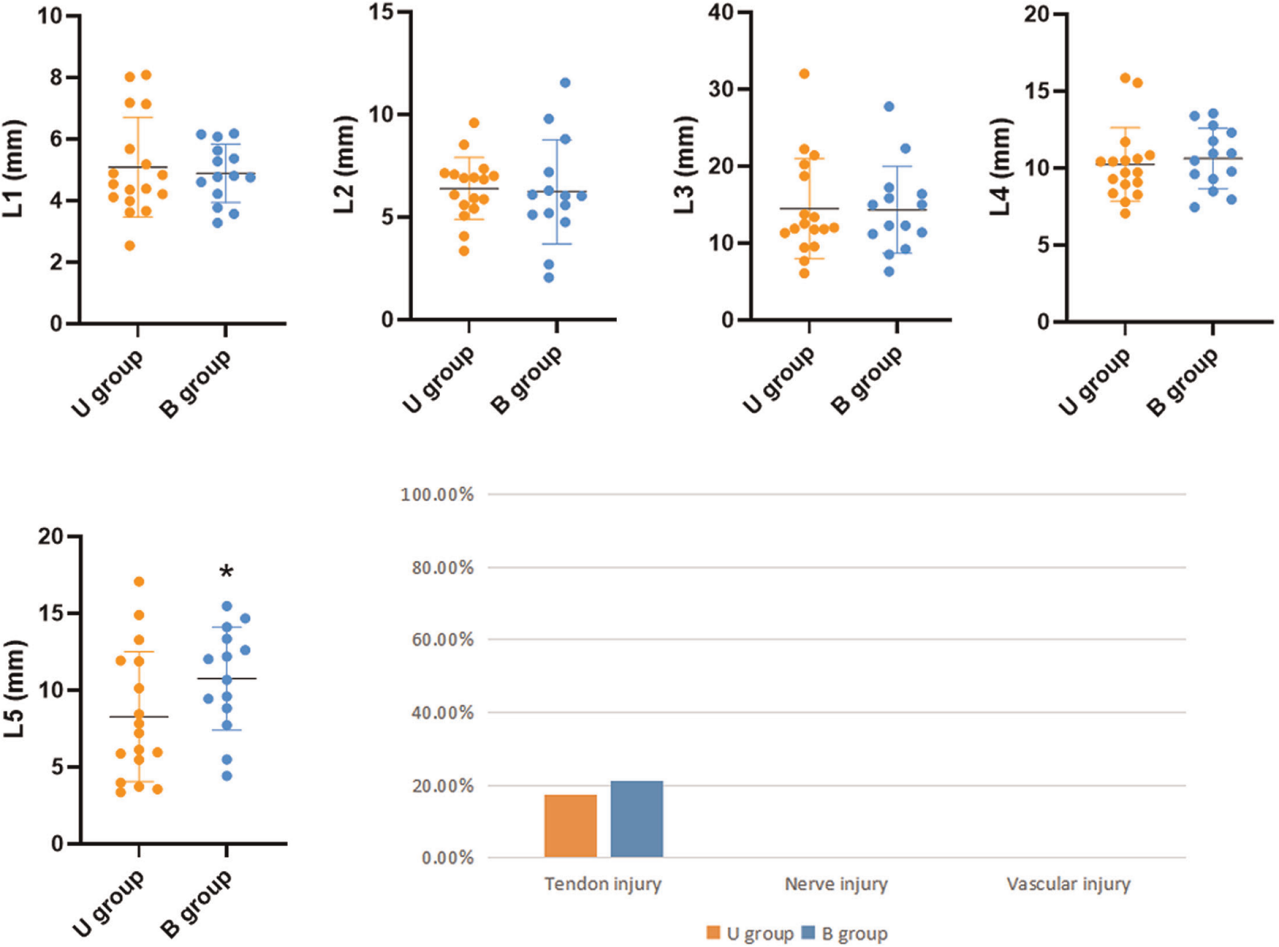
Distance of the incision from nerves and blood vessels and injury rate.

A learning curve shows the comparison of the ratio of releasing between Ultrasonic guidance operation (U group) and traditional blind operation (B group) (Figure 5). Ultrasonic guidance can provide a reasonable success rate of acupotomy operations in the early stage. After the operation of 7 specimens (14 wrists), the blind operation obtained a stable successful operation. In the U group, the three cases were missed, the needle tip punctured under the tendon sheath, causing slight scratches on the surface of the tendon. In group B, 6 cases were missed, three cases were punctured under the tendon sheath, one case lateral offset, and 2 cases were punctured between the skin and tendon sheath. The success rate of U group (17/20, 85%) is better than group B (14/20, 70%), (*P *< 0.05). No vascular or nerve injury was found in either procedure, and 3 cases with tendon injuries (slight scratches on the surface of the tendon) were found in both groups. Due to the ultrasound probe's placement, the ultrasonic group L5 cut trace is closer to the snuff nest. The other distance that acupotomy incision to the blood vessels and nerves has no statistically significant difference between U group and B group (*P* > 0.05) (Figure 6).

**Figure 7:**
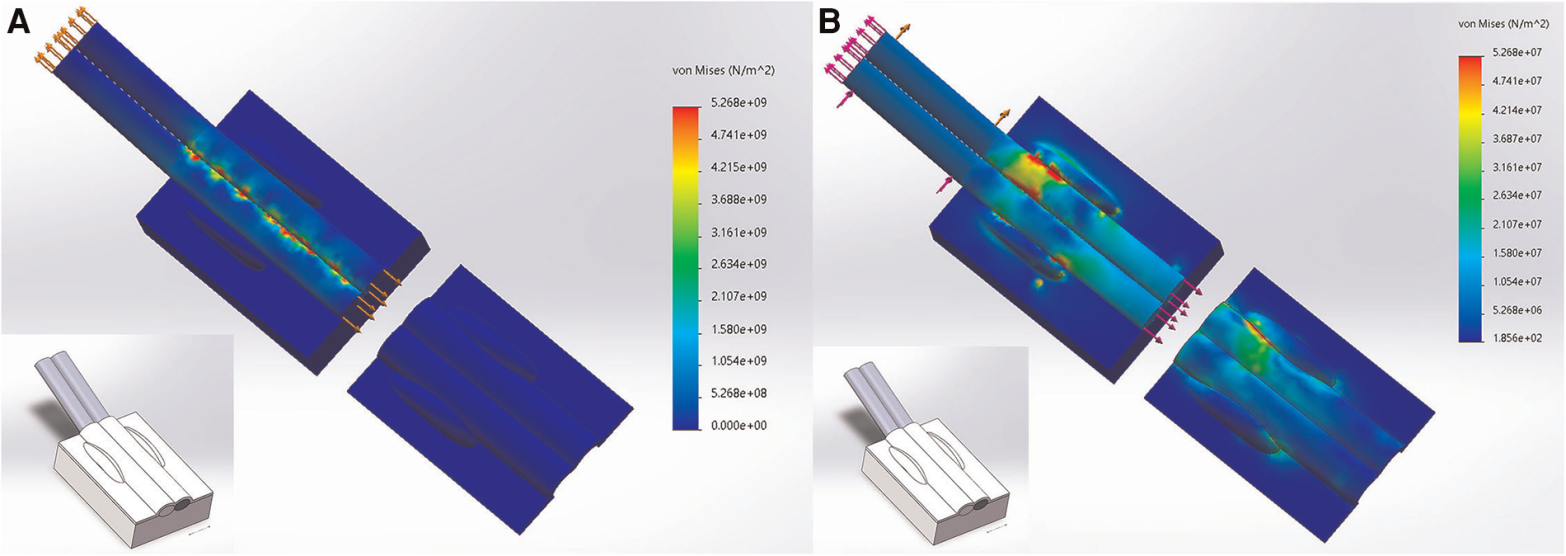
Finite element analysis of radial styloid process area. (**A**) the tendon is subjected to longitudinal traction; (**B**) the tendon is subjected to longitudinal and oblique traction.

A preliminary finite element analysis result shows (Figure 7) that when simulating the thumb's motion in the upright position of the wrist, the tendon is subjected to longitudinal traction, and the force is mainly between the tendons, forming friction rubs between the tendons. When simulating the force of wrist flexion, the tendon is subjected to longitudinal and oblique traction, and the force is mainly on the tendon and tendon sheath between the bony ridge.

**Figure 8:**
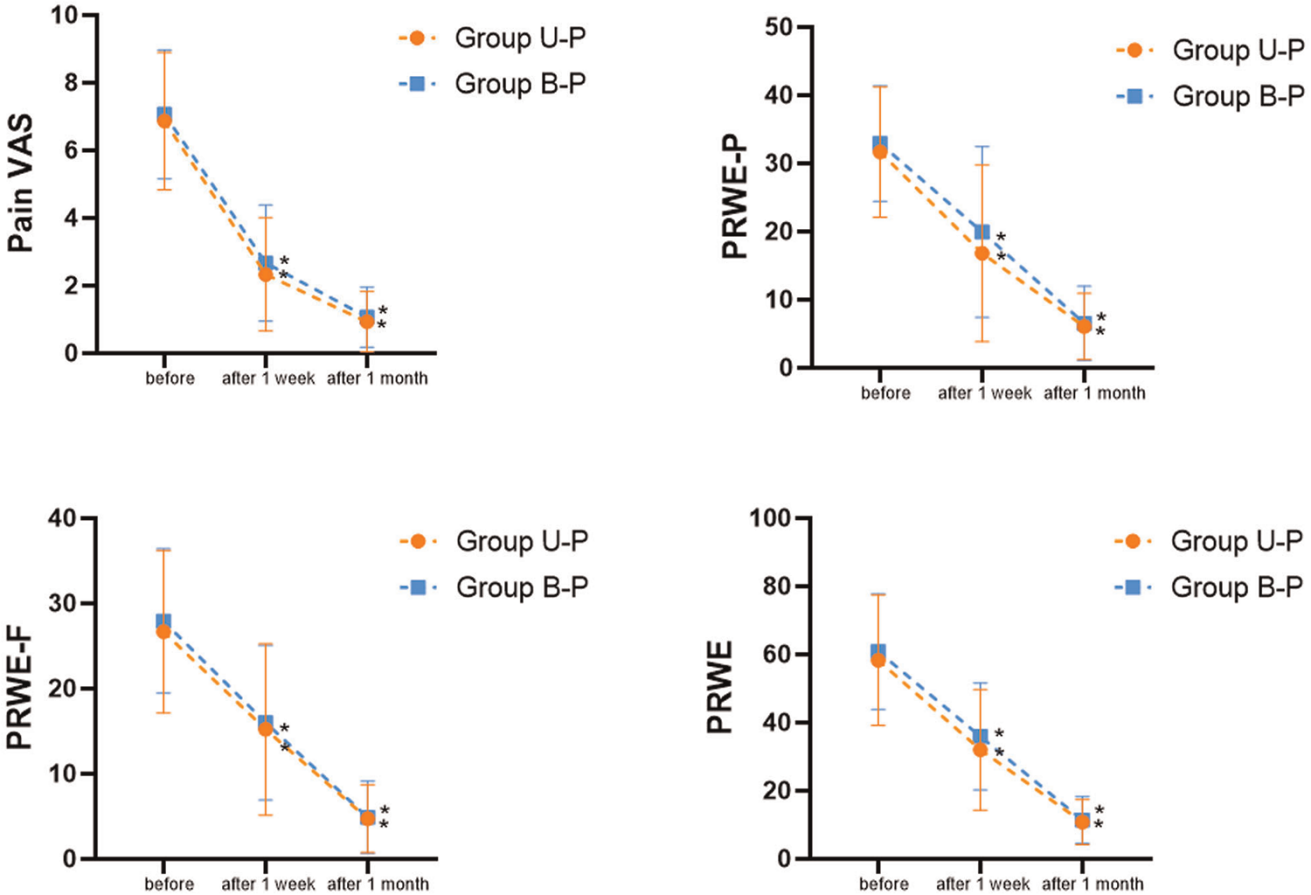
Comparison of the effects of ultrasound-guided acupotomy (group U-P) and blind acupotomy therapy (group B-P) in dQD patients. * compare to before (*P* < 0.05).

A total of 30 patients were included in the study; there was no statistically significant difference in gender, age, course of the disease, and affected side between the two groups. In this prospective study, both U-P group and B-P group achieved better improvements in pain and function; pain VAS, PRWE-P, PRWE-F, and PRWE decreased after treatment in one week and one month compared to before (*P* < 0.05), and the results with not statistically significant between U-P group and B-P group (*P* > 0.05) (Figure 8).

## Discussion

In this study, we evaluated the safety and released efficiency of acupotomy percutaneous release in the first extensor tendon compartment, with ultrasound-guided or blind techniques to treat dQS. Those two techniques both have well success rates in ultrasound-guided technique (85%) and blind technique (70%) in the cadaver study. Percutaneous release technology can change the structure of the sore site through minimally invasive incisions to reduce tendon and nerve entrapment (17); it can also avoid the risk of skin hypopigmentation (18) and tendon rupture caused by repeated steroid injections (19). Compared with the open procedure, the percutaneous technique is more cost-effective because it can be performed in the office, and offers the advantage of being less invasive, which decreases the risk for infection, scar tissue formation (20). According to one research, individuals who receive ultrasound-guided percutaneous release can resume regular activities 4.1 days following the procedure, instead of 17.8 days with an open approach (21). The percutaneous release technique has been relatively mature in trigger finger and carpal tunnel syndrome but has not been widely reported in dQD (22).

The learning curve in our experiments also indicated that ultrasound guidance could improve the success rate (85%) of beginners in dQD treatment compared with traditional blind manipulation (70%), increase the accurate positioning of the tendon sheath and reduce the penetration or deviation caused by blind operations. In the treatment of dQD, ultrasound can identify the intercompartmental septum and increase the success rate of injection. A systematic review of ultrasound research reported that, the prevalence of an intercompartmental septum in dQD patients (67%) is significantly greater than in the general population (35%), ultrasound-guided corticosteroid injections were more accurate than manual injections (90% vs. 40%), and to confer better treatment outcomes (100% vs. 83% success rates) (23).

**Figure 9:**
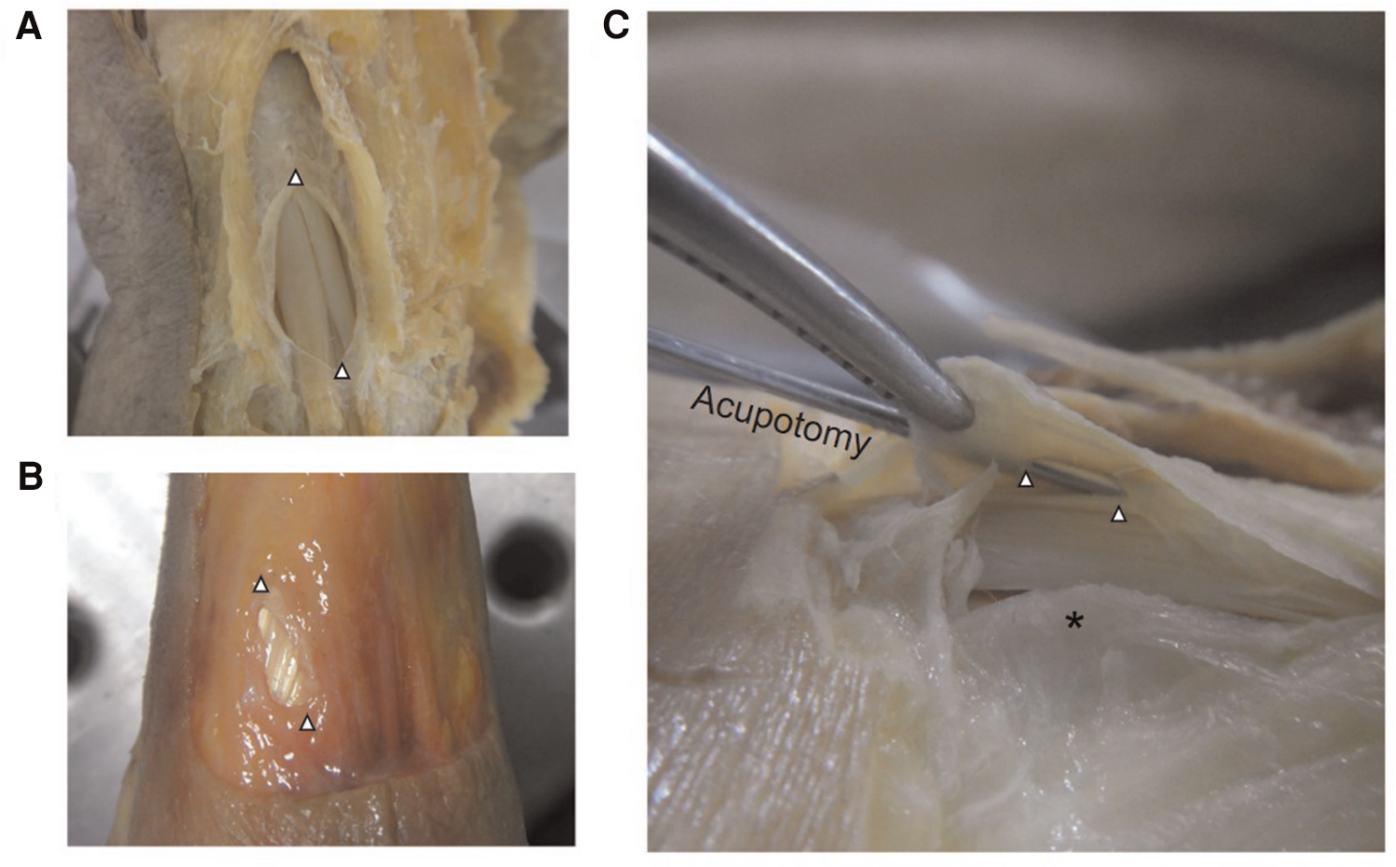
Acupotomy incisions in fixed (**A**) and fresh (**B**) specimens, the incision is widened with tweezers; bony ridge at the radial styloid process (**C**). △ acupotomy incisions; * bony ridge.

We put forward a different point of view from other studies (9–11). Due to two volar bony ridges in the radial styloid process (24), the extensor retinaculum fixes the two tendons here, and the narrow area formed by the bony bulge and tendon sheath enlarges the stress of tendon. A 0.5–1 cm incision of the tendon sheath between the bony ridges of the radial styloid process is an effective decompression strategy (Figure 9). Our hypothesis was validated in dQD patients with their pain areas between two bony ridges and the model was also validated in biomechanics. The tendon comes out with friction when the wrist is upright, and when the wrist is flexed, the tendon and tendon sheath is stressed in the bone ridges. A study found that the bony ridges at the radial styloid process can form a deep or flat the extensor groove, in the deep extensor groove, which was separated into two sub-grooves by a thin bony ridge, accounted for 63.73%, which further aggravates the narrowing of first extensor tendon compartment (25).

According to our clinic experience, the percutaneous release technology performed in DQD patients is safe and feasible. We further verify the ultrasonic-guidance and blind acupotomy percutaneous release technology through the prospective clinical study. Both operations has a good treatment effect on DQD patients by the doctors with skilled ultrasound guidance and acupotomy technology. A study reported cutting on the surface of the tendon sheath, a 90° rotation of the needle to orient the bevel perpendicular to the fibres being cut to release an intertendinous septum was required (10). Although there has not been reported whether release an intertendinous septum in dQD patients can achieve better results, the full-length release in the first extensor tendon compartment is not needed because it will cause tendon subluxation (26, 27). We demonstrated that releasing the tendon sheath between the bone ridges can depressurize the tendons. If the percutaneous release is unsuccessful, a second percutaneous release or an open procedure can be considered.

## Limitation

However, since we are the acupuncture department, there might be biased towards mild dQD patients in source, so the technique we reported still needs further validation for refractory dQS in orthopedics or pain department. Based on the current prospective study, a larger sample size of dQD patients with more than 3 months follow-up need to be performed in the next step.

## Conclusions

Both traditional and US-guided percutaneous release by acupotomy of the first extensor tendon compartment can be performed for all wrists. US-guided techniques can improve the success rate during acupotomy operations, especially for beginners and follows.

## Data Availability

The original contributions presented in the study are included in the article/Supplementary Materials, further inquiries can be directed to the corresponding author/s.

## References

[1] Kay NR. De Quervain's Disease. Changing pathology or changing perception? J Hand Surg Br. (2000) 25(1):65–9. 10.1054/jhsb.1999.0277 10763728

[2] Abate M Silbernagel KG Siljeholm C Di Iorio A De Amicis D Salini V Pathogenesis of tendinopathies: inflammation or degeneration? Arthritis Res Ther. (2009) 11(3):235. 10.1186/ar2723 19591655 PMC2714139

[3] Pensak MJ Bayron J Wolf JM. Current treatment of de Quervain tendinopathy. J Hand Surg-Am. (2013) 38(11):2247–9. 10.1016/j.jhsa.2013.06.003 23890846

[4] Abdoli A Hashemizadeh AS Jalil AS. Comparing the corticosteroid injection and A1 pulley percutaneous release in treatment of trigger finger: a clinical trial. J Hand Surg-Asian Pac. (2021) 26(2):207–13. 10.1142/S2424835521500193 33928857

[5] Mittal N Sangha H Flannery J Robinson LR Agur A. Ultrasound-guided incisionless carpal tunnel release using a hook knife: a cadaveric study. PM&R. (2019) 11(10):1101–6. 10.1002/pmrj.12118 30734506

[6] Guo D Kliot M McCool L Senk A Tonkin B Guo D. Percutaneous cubital tunnel release with a dissection thread: a cadaveric study. J Hand Surg-Eur. (2019) 44(9):920–4. 10.1177/1753193419856591 31189372

[7] Spies CK Muller LP Skouras E Bassemir D Hahn P Unglaub F. Percutaneous needle aponeurotomy for Dupuytren's Disease. Oper Orthopade Traumatol. (2016) 28(1):12–9. 10.1007/s00064-015-0417-5 26303259

[8] Nikolaou VS Malahias MA Kaseta MK Sourlas I Babis GC. Comparative clinical study of ultrasound-guided A1 pulley release vs open surgical intervention in the treatment of trigger finger. World J Orthop. (2017) 8(2):163–9. 10.5312/wjo.v8.i2.163 28251067 PMC5314146

[9] Gulec A Turkmen F Toker S Acar MA. Percutaneous release of the first dorsal extensor compartment: a cadaver study. PRS-Glob Open. (2016) 4(10):e1022. 10.1097/GOX.0000000000001022 PMC5096515 27826460

[10] Lapegue F Andre A Pasquier BE Akakpo EJ Laumonerie P Chiavassa-Gandois H US-guided percutaneous release of the first extensor tendon compartment using a 21-gauge needle in de Quervain's Disease: a prospective study of 35 cases. Eur Radiol. (2018) 28(9):3977–85. 10.1007/s00330-018-5387-1 29619521

[11] Croutzet P Guinand R Mares O Apard T Candelier G David I. Ultrasound-Guided de Quervain's Tendon release, feasibility, and first outcomes. J Wrist Surg. (2019) 8(6):513–9. 10.1055/s-0039-1678688 31815068 PMC6892647

[12] Yoon SH Kim YS Jo HG Kwon CY. Current usage of terminologies related to acupotomy: a literature research and standardization suggestion. Chin J Integr Med (2019) 25(2):147–50. 10.1007/s11655-018-3015-1 30328566

[13] Kwon CY Yoon SH Lee B. Clinical effectiveness and safety of acupotomy: an overview of systematic reviews. Complement Ther Clin Pract (2019) 36:142–52. 10.1016/j.ctcp.2019.07.002 31383431

[14] Henderson J Thoreson A Yoshii Y Zhao KD Amadio PC An KN. Finite element model of subsynovial connective tissue deformation due to tendon excursion in the human carpal tunnel. J Biomech. (2011) 44(1):150–5. 10.1016/j.jbiomech.2010.09.001 20887993 PMC3003739

[15] Main EK Goetz JE Rudert MJ Goreham-Voss CM Brown TD. Apparent transverse compressive material properties of the digital flexor tendons and the median nerve in the carpal tunnel. J Biomech. (2011) 44(5):863–8. 10.1016/j.jbiomech.2010.12.005 21194695 PMC3048925

[16] Sugiura S Matsuura Y Suzuki T Nishikawa S Toyooka T Ohtori S. Biomechanical assessment of the first dorsal compartment of the wrist: a fresh cadaver study with relevance to de Quervain's Disease. Clin Anat (2022) 35(8):1058–63. 10.1002/ca.23872 35434856

[17] Mansour J Ghanimeh J Ghersi A Moutinot B Coulomb R Kouyoumdjian P Percutaneous ultrasound-guided ulnar nerve release technique compared to open technique: a cadaveric study. SICOT-J. (2022) 8:40. 10.1051/sicotj/2022041 36155647 PMC9511962

[18] Milani C Lin C. Proximal linear extension of skin hypopigmentation after ultrasound-guided corticosteroid injection for de quervain tenosynovitis: a case presentation. PM&R. (2018) 10(8):873–6. 10.1016/j.pmrj.2018.01.001 29355747

[19] Nguyen ML F N. Jones: rupture of both the abductor pollicis longus and extensor pollicis brevis tendons after steroid injection for de quervain tenosynovitis. Plast Reconstr Surg. (2012) 129(5):883e–6e. 10.1097/PRS.0b013e31824aa06d 22544145

[20] Xie P Zhang QH Zheng GZ Liu DZ Miao HG Zhang WF Stenosing tenosynovitis: evaluation of percutaneous release with a specially designed needle vs. open surgery. Orthopade. (2019) 48(3):202–6. 10.1007/s00132-018-03676-4 30623237

[21] Brozovich N Agrawal D Reddy G. A critical appraisal of adult trigger finger: pathophysiology, treatment, and future outlook. PRS-Glob open. (2019) 7(8):e2360. 10.1097/GOX.0000000000002360 PMC6756654 31592381

[22] Moungondo F Feipel V. Percutaneous sonographically guided release of carpal tunnel and trigger finger: biomechanics, clinical results, technical developments. Hand Clin. (2022) 38(1):91–100. 10.1016/j.hcl.2021.08.010 34802613

[23] Abi-Rafeh J Mojtahed JM Kazan R Alabdulkarim A Boily M Thibaudeau S. Utility of ultrasonography and significance of surgical anatomy in the management of de quervain disease: a systematic review and meta-analysis. Plast Reconstr Surg. (2022) 149(2):420–34. 10.1097/PRS.0000000000008792 35077418

[24] Collins ED. Radial ridge excision for symptomatic volar tendon subluxation following de Quervain's Release. Tech Hand Up Extrem Surg. (2014) 18(3):143–5. 10.1097/BTH.0000000000000054 24977493

[25] Xiao L Li YK Ye GH Yang XW. Variations in the extensor grooves on the radial styloid process in Chinese population. Surg Radiol Anat. (2013) 35(1):49–53. 10.1007/s00276-012-0995-y 22744308

[26] Khurana A Agarwal P Gupta SC Malik K Jain V. Pulley reconstruction following surgical release of DC1 pulley in De Quervain's Tenosynovitis: surgical technique and case series. Arch Bone Jt Surg. (2022) 10(5):459–65. 10.22038/ABJS.2021.58872.2913 35755793 PMC9194712

[27] Renson D Mermuys K Vanmierlo B Bonte F Van Hoonacker P Kerckhove D Goubau: pulley reconstruction for symptomatic instability of the tendons of the first extensor compartment following de Quervain's Release. J Wrist Surg. (2018) 7(1):31–7. 10.1055/s-0037-1603686 29383273 PMC5788753

